# 1928. Trends In Incidence of Carbapenem-Resistant Enterobacterales (CRE) In Seven US sites, 2016─2020

**DOI:** 10.1093/ofid/ofad500.088

**Published:** 2023-11-27

**Authors:** Nadezhda Duffy, Rongxia Li, Christopher A Czaja, Helen Johnston, Sarah J Janelle, Jesse T Jacob, Gillian Smith, Lucy E Wilson, Elisabeth Vaeth, Ghinwa Dumyati, Rebecca Tsay, Christopher Wilson, Daniel Muleta, Jacquelyn Mounsey, Ruth Lynfield, Sean O’Malley, Paula S Vagnone, Rebecca Pierce, P Maureen Cassidy, Heather Hertzel, Sandra N Bulens, Julian E Grass, Alice Guh

**Affiliations:** CDC, Atlanta, Georgia; CDC, Atlanta, Georgia; Colorado Department of Public Health and Environment, Denver, Colorado; Colorado Department of Public Health, Denver, Colorado; Colorado EIP, Denver, Colorado; Emory University School of Medicine, Atlanta, GA; Georgia Emerging Infections Program, Atlanta, GA; Foundation for Atlanta Veterans Education and Research, Decatur, GA; Atlanta Veterans Affairs Medical Center, Decatur, GA, Atlanta, Georgia; University of Maryland Baltimore County, Baltimore, Maryland; Maryland Department of Health, Baltimore, Maryland, Baltimore, Maryland; New York Emerging Infections Program and University of Rochester Medical Center, Rochester, New York; New York Rochester Emerging Infections Program at the University of Rochester Medical Center, Rochester, NY; Tennessee Department of Health, Nashville, Tennessee; Tennessee Department of Health, Nashville TN, Antioch, Tennessee; TN EIP, Nashville, Tennessee; Minnesota Department of Health, St. Paul, MN; MN EIP, Minneapolis, Minnesota; MN EIP, Minneapolis, Minnesota; Oregon Health Authority, Portlant, Oregon; OR EIP, Portland, Oregon; OR EIP, Portland, Oregon; CDC, Atlanta, Georgia; CDC, Atlanta, Georgia; CDC, Atlanta, Georgia

## Abstract

**Background:**

Preventing CRE spread is a U.S. public health priority. We described changes in 2016─2020 CRE incidence rates in 7 U.S. sites that conduct population-based CRE surveillance for the Centers for Disease Control and Prevention’s Emerging Infections Program.

**Methods:**

An incident CRE case from 2016 onwards was defined as the 1^st^ isolation of *Escherichia coli, Klebsiella* spp., *or Enterobacter* spp. resistant to ≥1 carbapenem from a sterile site or urine in a surveillance area resident in a 30-day period. We reviewed medical records to classify cases as hospital-onset (HO) if the culture was obtained >3 days after hospital admission; healthcare-associated, community-onset (HACO) if the culture was obtained in a non-hospital setting or < 3 days after hospital admission in a person with healthcare exposures in the prior year; and community-associated (CA) if there were no healthcare risk factors. We calculated incidence rates using Census data. We used Poisson mixed effects regression models to perform 2016─2020 trend analyses, adjusting for sex, race/ethnicity, and age. We compared adjusted incidence rates between 2016 and subsequent years using incidence rate ratios (RR) and 95% confidence intervals (CI). We also repeated the analysis using a pre-2016 CRE surveillance case definition that was more specific for carbapenemase-producing CRE and required carbapenem nonsusceptibility (excluding ertapenem) and third-generation cephalosporin resistance.

**Results:**

Of 4996 CRE cases, 62% were HACO, 25% CA, and 14% HO. The crude CRE incidence rate per 100,000 was 7.51 in 2016 and 6.08 in 2020 and was highest for HACO, followed by CA and HO (Figure 1). Compared to 2016, the adjusted overall CRE incidence rate declined since 2018, with a decrease of 24% (RR 0.76; 95% CI: 0.70−0.83) in 2020 (Figure 2). HACO and CA CRE rates significantly decreased in 2020, but the HO rate did not. Similar trends were seen using the other case definition, except the decline in CA CRE rate since 2016 was not significant (Figure 3).

**Figure 1.**
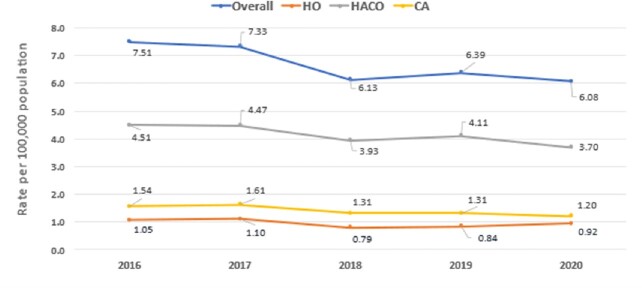
Crude CRE incidence rates, overall and by epidemiologic class, 2016─2020* *Current CRE surveillance case definition (from 2016 onwards) applied to all years

**Figure 2.**
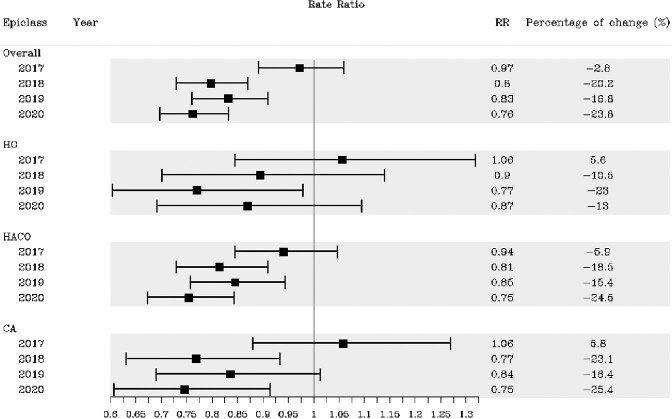
Adjusted rate ratios with 95% confidence intervals comparing annual 2017-2020 CRE incidence rates to the 2016 CRE incidence rate, using the current surveillance case definition* * Current CRE surveillance case definition (from 2016 onwards) applied to all years starting in 2016

**Figure 3.**
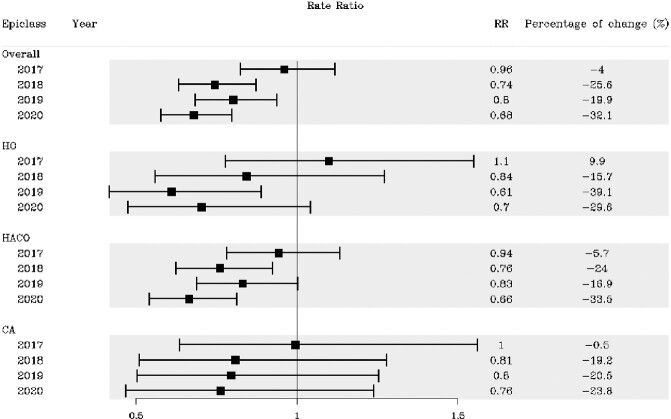
Adjusted rate ratios with 95% confidence intervals comparing annual 2017-2020 CRE incidence rates to the 2016 CRE incidence rate, using pre-2016 surveillance case definition* * Pre-2016 CRE surveillance case definition applied to all years starting in 2016

**Conclusion:**

Adjusted CRE incidence rates declined from 2016 to 2020 using current and prior case definitions but changes over time varied by epidemiologic class. Continued surveillance and effective control strategies are needed to prevent CRE in all settings.

**Disclosures:**

**Ghinwa Dumyati, MD**, Pfizer: Grant/Research Support **Rebecca Tsay, MPH**, CDC: Grant/Research Support

